# GINS1通过激活Notch/PI3K/AKT/mTORC1信号通路增强肺腺癌细胞糖酵解、增殖和转移

**DOI:** 10.3779/j.issn.1009-3419.2024.101.27

**Published:** 2024-10-20

**Authors:** Yishan HUO, Xiaohui XU, Xiumin MA, Yangchun FENG

**Affiliations:** 830011 乌鲁木齐，新疆医科大学附属肿瘤医院医学检验中心; Medical Laboratory Center, the Affiliated Cancer Hospital of Xinjiang Medical University, Urumqi 830011, China

**Keywords:** 肺腺癌, GINS1, Notch, 糖酵解, 增殖, 转移, Lung adenocarcinoma, GINS1, Notch, Glycolysis, Proliferation, Metastasis

## Abstract

**背景与目的:**

肺癌是所有癌症类型中占比最大的一种，其中肺腺癌（lung adenocarcinoma, LUAD）占肺癌患者的一半以上。目前，转移性LUAD患者5年生存率仍然较低，迫切需要新型生物标志物作为靶向治疗的靶点。Go-Ichi-Ni-San1（GINS1）是GINS家族的重要成员，与人类恶性肿瘤的发生和发展密切相关。本研究旨在探究GINS1在LUAD细胞糖酵解、增殖和转移过程中的作用及相关分子机制。

**方法:**

通过生物信息学分析GINS1在LUAD患者和健康人群中的表达差异。通过免疫组织化学染色和Western blot检测GINS1在LUAD组织和癌旁组织中的表达水平，采用Western blot和实时荧光定量聚合酶链式反应（real-time fluorescence quantitative polymerase chain reaction, qRT-PCR）检测GINS1在LUAD细胞系A549、SK-LU-1、Calu-3、H1299和人正常支气管上皮细胞系BEAS-2B中的表达水平。通过慢病毒转染细胞的方法构建稳定敲低GINS1的A549细胞株（shGINS1-A549）及其阴性对照细胞株（shGINS1-NC-A549）、稳定过表达GINS1的H1299细胞株（GINS1-OE-H1299）及其阴性对照细胞株（GINS1-OENC-H1299）。通过集落形成试验检测细胞增殖能力，划痕试验检测细胞迁移能力，Transwell试验检测细胞侵袭能力，试剂盒检测细胞对葡萄糖的消耗量以及乳酸的产量，Western blot检测糖酵解相关蛋白、Notch信号通路蛋白和磷脂酰肌醇-3-激酶（phosphatidylinositol-3-kinase, PI3K）/蛋白激酶B（protein kinase B, AKT）和哺乳动物雷帕霉素蛋白（mammalian target of rapamycin, mTOR）信号通路蛋白表达水平。向shGINS1-A549组细胞加入Notch受体激动剂Jagged1，向GINS1-OE-H1299组细胞加入Notch受体抑制剂LY3039478，进行回复试验。

**结果:**

GINS1在LUAD患者、组织和细胞系中均表达上调，并与患者总生存期有关（P<0.05）。敲低GINS1后，A549细胞的增殖、迁移和侵袭能力均受到显著抑制（P<0.05）；过表达GINS1则显著增强了H1299细胞的增殖、迁移和侵袭能力（P<0.05）。此外，敲低GINS1导致A549细胞对葡萄糖的消耗量减少，乳酸产量减少，糖酵解相关蛋白表达水平降低（P<0.05）；过表达GINS1则增强了H1299细胞糖酵解水平（P<0.05）。GINS1敲低导致A549细胞Notch1、Notch3、p-PI3K、p-AKT和p-mTORC1（Ser2448）蛋白表达水平显著降低（P<0.05），而PI3K、AKT、mTOR、p-mTORC2（Ser2481）蛋白表达水平无显著变化（P>0.05）；GINS1过表达则升高了H1299细胞Notch1、Notch3、PI3K/AKT/mTORC1通路磷酸化蛋白水平（P<0.05）。Jagged1显著逆转了由于GINS1敲低导致的A549细胞糖酵解、增殖和转移活性抑制（P<0.05）；LY3039478显著抑制了由于GINS1过表达诱导的H1299细胞糖酵解、增殖和转移活性增强（P<0.05）。

**结论:**

GINS1的表达通过促进Notch1、Notch3受体表达水平升高，进而磷酸化激活下游PI3K/AKT/mTORC1信号通路，增强LUAD细胞糖酵解、增殖和转移。

## Abstract (en)

**Background and objective:**

Lung cancer is the most common type of cancer, accounting for more than half of all cancer cases, with lung adenocarcinoma (LUAD) representing over half of lung cancer patients. Currently, the 5-year survival rate for metastatic LUAD patients remains low and there is an urgent need for new biomarkers as targets for targeted therapy. Go-Ichi-Ni-San 1 (GINS1), an important member of the GINS family, is closely related to the occurrence and development of human malignant tumors. This study aims to explore the role of GINS1 in glycolysis, proliferation, and metastasis of LUAD cells and the related molecular mechanisms.

**Methods:**

The expression of GINS1 was analysed using bioinformatics between LUAD patients and healthy controls. The expression levels of GINS1 in LUAD and adjacent tissues were detected by immunohistochemistry and Western blot. Western blot and real-time fluorescence quantitative polymerase chain reaction (qRT-PCR) were used to detect the expression of GINS1 in LUAD cell lines A549, SK-LU-1, Calu-3, H1299 and BEAS-2B. Stably knockdown GINS1 in A549 cells and its negative control cell line, as well as stably overexpress GINS1 in H1299 cells and its negative control cell line, were constructed by lentiviral transduction. Colony formation test was used to detect cell proliferation. Scratch test was used to detect cell migration. Transwell test was used to detect cell invasion, and the test kits were used to detect glucose consumption and lactate production. The expression levels of glycolysis-related proteins, Notch signaling pathway proteins and phosphatidylinositol-3-kinase/protein kinase B/mammalian target of rapamycin (PI3K/AKT/mTOR) signaling pathway proteins were detected by Western blot. The Notch receptor agonist Jagged1 was added to cells from the shGINS1-A549 group and the Notch receptor inhibitor LY3039478 was added to cells from the GINS1-OE-H1299 group for the regression assay.

**Results:**

The expression of GINS1 was up-regulated in LUAD patients, tissues and cell lines, and correlated with overall survival (P<0.05). Knockdown of GINS1 significantly inhibited the proliferation, migration and invasion of A549 cells (P<0.05), while overexpression of GINS1 significantly enhanced the proliferation, migration and invasion of H1299 cells (P<0.05). Furthermore, knockdown of GINS1 resulted in reduced glucose consumption, reduced lactate production, and reduced expression levels of glycolytic-related proteins in A549 cells (P<0.05); overexpression of GINS1 enhanced glycolytic level in H1299 cells (P<0.05). The expression levels of Notch1, Notch3, phosphorylated-PI3K (p-PI3K), phosphorylated-AKT (p-AKT) and phosphorylated-mTORC1 (Ser2448)[p-mTORC1 (Ser2448)] in A549 cells were significantly decreased by GINS1 knockdown (P<0.05), while the expression levels of PI3K, AKT, mTOR and p-mTORC2 (Ser2481) were not significantly changed (P>0.05). Overexpression of GINS1 increased the levels of Notch1, Notch3 and PI3K/AKT/mTORC1 pathway phosphorylated proteins in H1299 cells (P<0.05). Jagged1 significantly reversed the inhibition of glycolysis, proliferation and metastasis induced by GINS1 knockdown in A549 cells (P<0.05), and LY3039478 significantly inhibited the enhancement of glycolysis, proliferation and metastasis induced by GINS1 overexpression in H1299 cells (P<0.05).

**Conclusion:**

The expression of GINS1 enhances the expression of Notch1 and Notch3 receptors, and then phosphorylates and activates the downstream PI3K/AKT/mTORC1 signaling pathway to enhance the glycolysis, proliferation and metastasis of LUAD cells.

肺癌是所有癌症类型中占比最大的一种，其中肺腺癌（lung adenocarcinoma, LUAD）占肺癌患者的一半以上^[1]^。诊疗水平的进步提高了LUAD患者的总生存期和生存质量，但转移性LUAD患者的5年生存率低于20%，肺癌的发病率和死亡率呈上升趋势^[2,3]^。因此，为了提高患者预后和生存率，迫切需要进一步发展针对LUAD患者特异的治疗靶点。

GINS1（Go-Ichi-Ni-San1）是GINS复合体家族的重要成员，其编码基因染色体定位为20p11.21^[4]^。GINS复合体与哺乳动物细胞中参与DNA复制的蛋白质结合，协同完成DNA复制过程^[5]^。GINS1是GINS复合体中的关键角色，在整个细胞周期中维持细胞增殖活性，未发生增殖的细胞内GINS1几乎不表达^[6]^。GINS1与多种人类恶性肿瘤进展相关^[7]^。比如，GINS1基因敲低能够显著抑制鼻咽癌细胞活性，导致肿瘤生长、迁移和侵袭行为减弱^[8]^。在胶质瘤中，GINS1能够通过影响细胞内去泛素化过程促进肿瘤细胞增殖和迁移^[9]^。GINS1还与肿瘤细胞耐药性的产生有关。据报道，GINS1通过促进人类肝癌细胞的癌症干细胞特性来诱导索拉非尼耐药性的产生^[10]^。GINS1在肺癌中也有着重要作用。比如，黎明^[11]^研究证实GINS1为非小细胞肺癌的鉴定标志物。然而，关于GINS1在LUAD进展过程中的作用和相关分子机制仍未完全阐明。本研究通过在不同LUAD细胞系中改变GINS1表达水平，揭示了GINS1在LUAD细胞糖酵解、增殖和转移过程中对磷脂酰肌醇-3-激酶（phosphatidylinositol-3-kinase, PI3K）/蛋白激酶B（protein kinase B, AKT）和哺乳动物雷帕霉素蛋白（mammalian target of rapamycin, mTOR）通路的调节作用。

## 1 材料和方法

### 1.1 细胞系、临床样本来源

人LUAD细胞系A549、SK-LU-1、Calu-3、H1299和人正常肺上皮细胞系BEAS-2B均购自武汉普诺赛生物公司。22例临床确诊LUAD患者病理组织切片来自新疆医科大学附属肿瘤医院，患者均为新确诊LUAD且之前未接受任何新辅助化疗。样本采集和实验方案经过新疆医科大学附属肿瘤医院伦理委员会审批通过（审批号：K-2023013），患者均签署知情同意书。

### 1.2 主要试剂

DMEM培养基、胎牛血清购自美国Gibco公司；嘌呤霉素、放射免疫沉淀法裂解缓冲液（radio immunoprecipitation assay lysisbuffer, RIPA）购自上海碧云天公司；ECL（enhanced chemiluminescent）超敏化学发光液购自美国Thermo Fisher公司；抗β-actin、GINS1、Notch1、Notch3抗体购自美国Abcam公司；抗磷酸果糖激酶1（phosphofructokinase-1, PFK1）、乳酸脱氢酶A（lactate dehydrogenase A, LDHA）、葡萄糖转运蛋白1（glucose transporter-1, GLUT1）、己糖激酶2（hexokinase 2, HK2）抗体购自美国Proteintech公司；抗PI3K、磷酸化PI3K（phosphorylated-PI3K, p-PI3K）、AKT、磷酸化AKT（phosphorylated-AKT, p-AKT）、mTOR、磷酸化mTOR复合物1（Ser2448）[phosphorylated-mTOR complex 1 (Ser2448)，p-mTORC1 (Ser2448)]、p-mTORC2（Ser2481）购自美国CST公司；羊抗兔、羊抗鼠二抗购自北京索莱宝公司；TRIzol试剂、ChamQ SYBR qPCR Master Mix试剂盒购自南京Vazyme公司；SuperScript试剂盒购自美国Thermo Fisher公司；葡萄糖购自美国Sigma公司；葡萄糖含量检测试剂盒购自沈阳万类生物公司，乳酸检测试剂盒购自南京建成生物公司；Jagged1多肽、LY3039478购自美国MCE公司。

### 1.3 方法

#### 1.3.1 细胞系和细胞培养

所有细胞系均使用含10%胎牛血清的DMEM培养基培养。细胞放置在含5% CO_2_的37^ o^C孵育箱内。每天观察细胞状态，根据细胞生长情况进行培养基更换。

#### 1.3.2 生物信息学分析

根据GEPIA2数据网站（http://gepia2.cancer-pku.cn/#index）数据分析GINS1基因在LUAD患者和健康人群中的表达差异，并得到统计分析图。

#### 1.3.3 慢病毒转染细胞

包含GINS1基因表达干扰序列（short hairpin RNA, shRNA）及其阴性对照（negative control, NC）序列（shNC-RNA）、GINS1基因过表达序列（overexpression, OE）及其阴性对照序列（OENC）的慢病毒载体均购自上海吉玛基因公司，按照制造商的说明进行慢病毒转染细胞。具体而言，将A549和H1299细胞分别按5000个/孔接种于24孔板内。24 h后加入慢病毒-培养基混合液，继续培养72 h。之后更换为含10 μg/mL嘌呤霉素的新鲜培养基，对细胞进行筛选。将得到的细胞株分组为：shGINS1-A549和shGINS1-NC-A549；GINS1-OE-H1299和GINS1-OENC-H1299。本研究所用到GINS1基因的3个shRNA序列见表1。

**表1:** GINS1基因shRNA序列

shRNA	Sequence (5’-3’)
shGINS1-#1	CCGGCAAGTTCTGGAGGAGATGAAACTCGAGTTTCATCTCCTCCAGAACTTGTTTTTT
shGINS1-#2	CCCGGCAGACAAGTTCTGGAGGAGATCTCGAGATCTCCTCCAGAACTTGTCTGTTTTT
shGINS1-#3	CCGGACCACTGTTCTCTGTTAAGAACTCGAGTTCTTAACAGAACAGTGTCTTTTTT

shRNA: short hairpin RNA; GINS1: Go-Ichi-Ni-San1.

#### 1.3.4 Western blot

对于细胞样本，先将细胞接种于6孔板内，待细胞长满后，用含磷酸酶抑制剂的RIPA裂解液裂解细胞获得蛋白样品。蛋白样品在金属浴中煮沸变性，然后通过聚丙烯酰胺凝胶电泳分离，并转移到聚偏二氟乙烯膜上。用5%脱脂牛奶封闭膜2 h，将膜与一抗在4 ^o^C下孵育过夜。然后，膜与二抗在室温下孵育1 h。最后，用ECL化学发光液对蛋白进行显影。对于组织样本，将组织切成小块，按每30 mg组织需100 μL裂解液的比例加入裂解液，然后组织匀浆在冷冻研磨机上充分研磨，结束后静置10 min，取上清液，后续操作同前。所有一抗进行1:1000稀释后使用，二抗进行1:5000稀释后使用。以β-actin作为内源性参照。

#### 1.3.5 实时荧光定量聚合酶链式反应（real-time fluorescence quantitative polymerase chain reaction, qRT-PCR）

使用TRIzol试剂提取细胞/组织总RNA。之后，使用SuperScript试剂盒对RNA进行逆转录，得到cDNA。通过ChamQ SYBR qPCR Master Mix试剂盒对cDNA进行qPCR。以甘油醛-3-磷酸脱氢酶（glyceraldehyde-3-phosphate dehydrogenase, GAPDH）基因表达水平作为对照，使用2^-ΔΔCt^法计算相对表达量。本研究qRT-PCR中各基因引物序列见表2。

**表2:** qRT-PCR反应各基因引物序列

Gene	Primer sequences (5’-3’)
GAPDH	F: TGTGGGCATCAATGGATTTGG R: ACACCATGTATTCCGGGTCAAT
GINS1	F: ACGAGGATGGACTCAGACAAG R: TGCAGCGTCGATTTCTTAACA

qRT-PCR: real-time fluorescence quantitative polymerase chain reaction; IHC: immunohistochemical staining; GAPDH: glyceraldehyde-3-phosphate dehydrogenase; F: forward; R: reverse.

#### 1.3.6 划痕试验

将细胞接种于6孔板内，待细胞汇合度达到70%，用无菌10 μL枪头对细胞进行划痕制造“缺口”。以无菌PBS轻轻洗涤3次，然后用无血清培养基继续培养细胞。分别在划痕第0 h和第48 h在显微镜下观察“缺口”面积变化，通过Image J软件对划痕愈合面积比例进行分析。

#### 1.3.7 集落形成试验

将处于对数生长期的细胞按200个/孔接种于6孔板内，均匀铺开，连续培养至肉眼可见明显的细胞集落形成。用4%多聚甲醛对细胞进行固定，用0.1%结晶紫溶液对细胞进行染色。观察并统计每孔细胞形成集落数量。

#### 1.3.8 Transwell试验

将处于对数生长期的细胞消化并离心，重悬于无血清培养基中，按1000个/孔的密度接种于铺有Matrigel胶的Transwell板（美国Corning公司）上室内，下室加入600 μL含15%胎牛血清的完全培养基。48 h后，以4%多聚甲醛对细胞进行固定，0.1%结晶紫溶液对细胞进行染色，在显微镜下观察并统计发生侵袭的细胞数量。

#### 1.3.9 免疫组织化学染色（immunohistochemical staining, IHC）

切片用乙醇水合，然后用澄清的二甲苯使其透明，包埋在石蜡中。然后用过氧化氢来抑制内源性过氧化物酶的功能。按照制造商的说明，孵育相应的一抗和二抗。使用倒置显微镜，每个载玻片以×100或×200的放大倍数进行检查。IHC结果由两位病理学专家独立进行评估。GINS1阳性表示为棕色，比较肿瘤组织和癌旁组织中免疫染色的阳性强度。

#### 1.3.10 葡萄糖、乳酸含量测定

收集各组细胞，按2000个/孔接种于96孔板内，给予各组细胞葡萄糖初始浓度均为5 mmol/L的培养体系（不含乳酸）进行培养。培养48 h后收集各组细胞培养上清，按试剂盒使用说明书进行葡萄糖或乳酸含量检测，在酶标仪上相应波长下测定吸光度值，并根据试剂盒说明书计算对应浓度。

### 1.4 统计学分析

所有实验均独立重复3次，使用GraphPad Prism 8.0软件评估处理数据。数据以均数±标准差表示。采用非配对t检验来比较两组数据的差异，采用单因素方差分析来比较三组数据之间的差异。以P<0.05作为差异有统计学意义的标准。

## 2 结果

### 2.1 GINS1在LUAD中表达上调

数据库检索结果显示，GINS1在LUAD患者中表达水平显著高于健康人群（图1A），并且与患者生存期总生存期有关（图1B）。为了验证数据库结果，本研究对收集的22例临床LUAD患者肿瘤组织和对应癌旁组织进行了IHC和Western blot检测，结果显示GINS1在LUAD组织表达上调（图1C-1D）。此外，Western blot和qRT-PCR结果显示，GINS1在LUAD细胞系中表达上调（图1E-1F）。

**图1:**
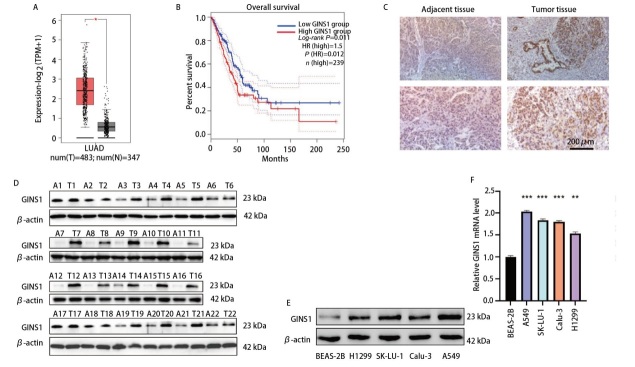
GINS1在LUAD中表达上调。A、B：通过GEPIA2数据库检索GINS1在LUAD患者和健康人群中的表达差异，并进一步分析GINS1表达水平与LUAD患者总生存期之间的关系；C、D：通过IHC和Western blot检测GINS1蛋白在LUAD患者肿瘤组织（n=22）和癌旁组织（n=22）中的表达水平；E、F：通过Western blot和qRT-PCR检测GINS1在4种LUAD细胞系和1种正常支气管上皮细胞系中的表达水平。*P<0.05；**P<0.01；***P<0.001。

### 2.2 GINS1敲低抑制LUAD细胞增殖、迁移和侵袭，过表达则相反

本研究通过慢病毒转染细胞的方法构建稳定敲低GINS1的A549细胞株（shGINS1-A549）及其阴性对照细胞株（shGINS1-NC-A549）、稳定过表达GINS1的H1299细胞株（GINS1-OE-H1299）及其阴性对照细胞株（GINS1-OENC-H1299）。如图2A-2B所示，3个shRNA均有效干扰了A549细胞内GINS1表达，并且以shGINS1-#2干扰效率最佳，shGINS1-#3干扰效率次之；而在H1299细胞内GINS1表达得到增强。随后，使用转染后细胞进行了集落形成试验、划痕试验以及Transwell试验。

**图2:**
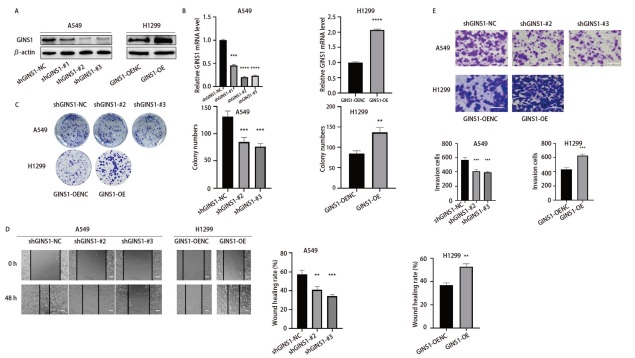
GINS1敲低抑制了LUAD细胞增殖、迁移和侵袭，过表达则相反。A、B：通过Western blot和qRT-PCR检测GINS1敲低或过表达效率；C：集落形成试验检测转染后A549和H1299细胞增殖活性变化；D：划痕试验检测转染后A549和H1299细胞迁移能力变化。标尺：400 μm；E：48 h Transwell试验检测转染后A549和H1299细胞侵袭能力变化。标尺：200 μm。**P<0.01；***P<0.001；****P<0.0001。

如图2C所示，在相同时间内，shGINS1-A549组细胞形成的集落数量显著少于shGINS1-NC-A549组细胞。在48 h划痕试验中，shGINS1-A549组细胞划痕“缺口”愈合面积比例显著少于shGINS1-NC-A549组细胞（图2D）。Transwell结果表明，48 h内shGINS1-A549组细胞发生侵袭行为的细胞数量显著少于shGINS1-NC-A549组细胞（图2E）。相反，与GINS1-OENC-H1299组细胞相比，GINS1-OE-H1299组细胞的增殖、迁移和侵袭活性均显著增强（图2C-2E）。

### 2.3 GINS1敲低抑制LUAD细胞糖酵解水平，过表达则相反

给予上述各组细胞相同葡萄糖初始浓度（5 mmol/L）的培养体系，培养24 h后检测了各组细胞培养体系内葡萄糖含量和乳酸含量。结果表明，培养24 h后，shGINS1-A549组细胞培养体系内葡萄糖含量高于shGINS1-NC-A549组细胞，乳酸产量低于shGINS1-NC-A549组（图3A）；GINS1-OE-H1299组细胞培养体系内葡萄糖含量低于GINS1-OENC-H1299组细胞，乳酸产量更高（图3B）。这说明GINS1的表达可能与LUAD细胞内糖酵解水平相关。PFK1、LDHA、GLUT1和HK2是调节糖酵解途径通量的关键蛋白。于是，本研究提取了各组细胞总蛋白进行Western blot分析，发现shGINS1-A549组细胞内上述糖酵解相关蛋白的表达水平均显著低于shGINS1-NC-A549组细胞，而GINS1-OE-H1299组细胞内PFK1、LDHA、GLUT1和HK2蛋白水平则显著高于GINS1-OENC-H1299组细胞（图3C），表明GINS1的表达水平与LUAD细胞糖酵解水平呈正相关。

**图3:**
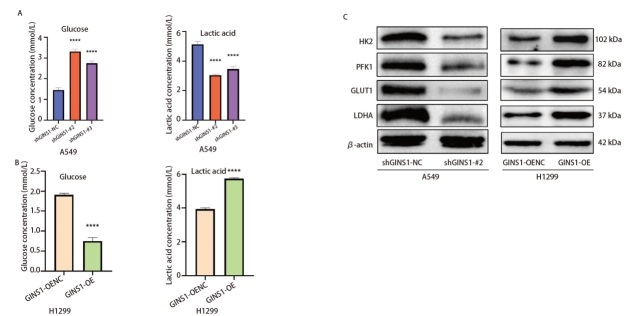
GINS1敲低抑制了LUAD细胞糖酵解水平，过表达则相反。A、B：使用葡萄糖含量检测试剂盒和乳酸含量检测试剂盒分别检测各组细胞培养体系中葡萄糖和乳酸含量；C：Western blot检测各组细胞糖酵解相关蛋白（LDHA、GLUT1、PFK1、HK2）表达水平。****P<0.0001。

### 2.4 GINS1与Notch/PI3K/AKT/mTORC1通路相关

Notch信号通路包括Notch1-4四种受体，据报道，Notch1和Notch3受体与LUAD进展相关^[12]^。Western blot结果显示，在shGINS1-A549组细胞内，Notch1和Notch3受体表达水平均显著低于shGINS1-NC-A549组细胞；而在GINS1-OE-H1299组细胞内，Notch1和Notch3受体表达水平均显著高于GINS1-OENC-H1299组细胞（图4A），说明GINS1表达水平的改变影响了LUAD细胞Notch1和Notch3受体表达水平。

**图4:**
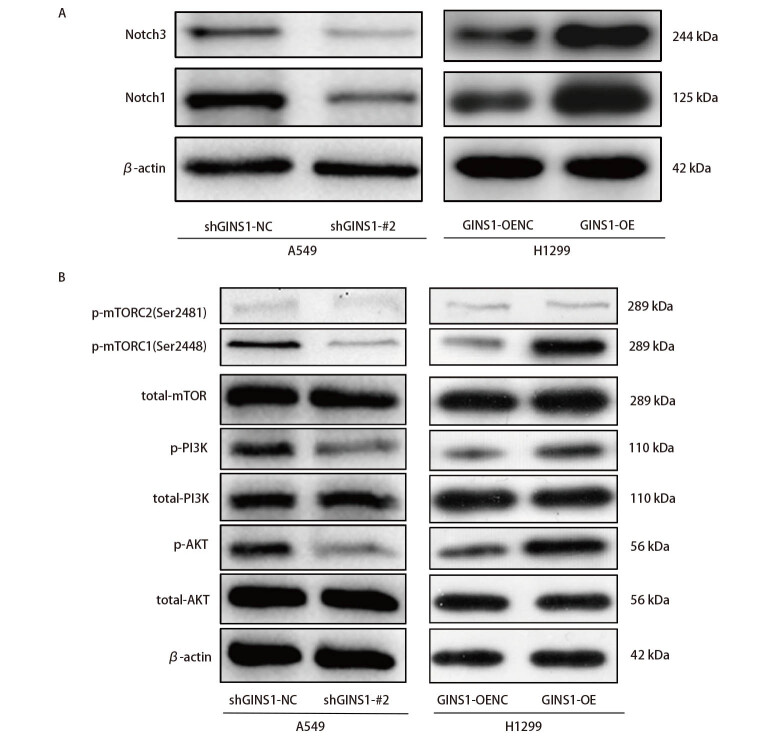
GINS1与Notch/PI3K/AKT/mTORC1通路相关。A：Western blot分析各组细胞内Notch1和Notch3受体表达水平变化；B：Western blot检测各组细胞内PI3K/AKT/mTOR通路蛋白及其磷酸化蛋白水平，Ser2448为mTORC1磷酸化位点，Ser2481为mTORC2磷酸化位点。

Notch通路能够调控激活其下游PI3K/AKT/mTOR通路，这种调控机制在LUAD的发生发展过程中有着重要作用^[12]^。Western blot结果显示，与shGINS1-NC-A549组细胞相比，shGINS1-A549组细胞内p-PI3K、p-AKT以及p-mTORC1（Ser2448）表达水平显著降低，而总PI3K、总AKT、总mTOR、p-mTORC2（Ser2481）表达水平无显著变化；与GINS1-OENC-H1299组细胞相比，GINS1-OE-H1299组细胞内p-PI3K、p-AKT以及p-mTORC1（Ser2448）表达水平显著升高，而总PI3K、总AKT、总mTOR、p-mTORC2（Ser2481）表达水平无显著变化（图4B）。

### 2.5 GINS1通过调节Notch/PI3K/AKT/mTORC1通路发挥促癌作用

为了验证GINS1在LUAD细胞中表现的促癌作用是由Notch/PI3K/AKT/mTORC1通路介导，分别向shGINS1-A549组细胞中加入Notch受体激动剂Jagged1多肽（20 ng/mL），向GINS1-OE-H1299组细胞中加入Notch受体抑制剂LY3039478（20 ng/mL）。如图5A所示，加入Jagged1后，shGINS1-A549组细胞形态饱满，增殖活性强，细胞密度增加；加入LY3039478后，GINS1-OE-H1299组细胞形态老化，增殖活性低，细胞密度减少。Western blot结果显示，加入Jagged1后，shGINS1-A549组细胞PI3K/AKT/mTORC1通路磷酸化水平升高，糖酵解水平升高；加入LY3039478后，GINS1-OE-H1299组细胞PI3K/AKT/mTORC1通路磷酸化水平降低，糖酵解水平降低（图5B）。

**图5:**
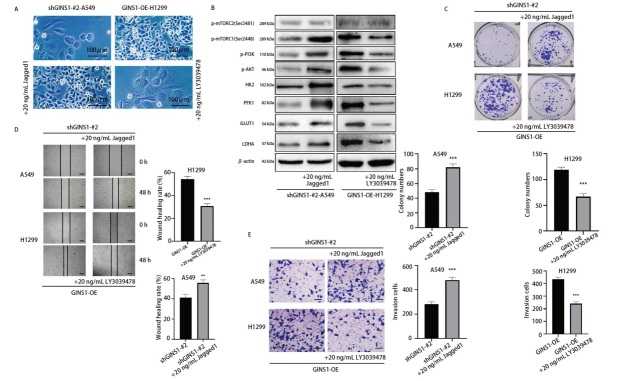
GINS1通过调节Notch/PI3K/AKT/mTORC1通路发挥促癌作用。A：加入Jagged1或LY3039478后，显微镜下观察细胞生长状态；B：Western blot检测各组细胞糖酵解相关蛋白、PI3K/AKT/mTOR通路磷酸化水平；C-E：集落形成试验、划痕试验、Transwell试验分别检测各组细胞在加入Jagged1或LY3039478后增殖、迁移、侵袭活性变化。图D标尺：400 μm；图E标尺：200 μm。**P<0.01；***P<0.001。

在集落形成试验中，Jagged1显著增加了shGINS1-A549组细胞的增殖活性；LY3039478则显著抑制了GINS1-OE-H1299组细胞增殖活性（图5C）。划痕试验和Transwell试验结果表明，Jagged1激活Notch/PI3K/AKT/mTORC1通路后显著增强了shGINS1-A549细胞的迁移和侵袭能力，而LY3039478抑制该通路则显著减弱了GINS1-OE-H1299细胞的迁移和侵袭能力（图5D-5E），说明GINS1对LUAD细胞增殖、迁移和侵袭行为的作用也是由Notch/PI3K/AKT/mTORC1通路介导的。

## 3 讨论

LUAD是肺部常见的恶性肿瘤，在全球发病率和死亡率常年居高不下。找到LUAD治疗特异性靶点，将有利于提高患者预后和生存期。肿瘤细胞分化程度低，增殖能力极强。研究^[13]^显示，GINS1高表达的细胞、组织和器官表现出较强的增殖或更新能力。有报道^[14]^提出，GINS1在肺癌中过表达，GINS1的缺失会影响肺癌细胞的细胞周期停滞和增殖。由于启动子甲基化程度高，导致GINS1在乳腺癌患者中高表达。在胶质瘤中，GINS1通过协助泛素特异性蛋白酶15去泛素化拓扑异构酶IIα来促进肿瘤细胞迁移和增殖^[9]^。另一方面，据报道^[15]^，GINS1还可帮助细胞毒性T淋巴细胞识别病毒抗原，以达到杀伤病毒的目的。

Notch信号是相邻细胞之间保守的通讯系统，影响多细胞生物发育过程中许多细胞命运的决定。异常Notch信号也与许多人类疾病的发生和进展有关^[16]^。PI3K/AKT/mTOR通路在调节细胞增殖、运动和存活方面起着关键作用。该通路上游信号的变化及其对下游信号的影响在不同病理过程中有着不同形式，这使得其在肿瘤的发生发展过程中同时起着促进/抑制作用^[17]^。代谢异常是肿瘤细胞的特征表现之一。糖酵解是细胞内重要的生物学活动，为细胞提供了活动能量，在肿瘤细胞中糖酵解水平往往表现异常。既往有研究^[18]^发现Notch信号能够通过PI3K/AKT通路介导细胞内糖酵解转换，以支持胚胎发育。进一步的研究^[19]^表明，Notch信号在胚胎发育过程中以依赖环境的方式调节糖酵解相关基因的表达。类似的是，另一项研究^[20]^表明，Notch信号通过直接调控参与糖酵解和三羧酸循环相关基因的表达来刺激细胞生长。泛素特异性肽酶24能够通过维持激酶PLK1激活Notch1受体，增强胃癌细胞有氧糖酵解和肿瘤进展^[21]^。在乳腺癌中，过度激活或低活性的Notch信号均增强了细胞糖酵解水平，糖酵解水平的升高极大增强了实体瘤细胞在缺氧环境中的生存能力^[22]^。本研究发现，GINS1敲低导致A549细胞糖酵解水平下降，而过表达GINS1则升高了H1299细胞糖酵解水平，提示GINS1的表达与LUAD细胞糖酵解途径存在一定联系。

Notch3受体与肿瘤的进展紧密相关，包括LUAD。比如，研究^[23]^发现，Notch3受体过表达与LUAD的酪氨酸激酶抑制剂耐药性相关。有研究^[24]^显示，Notch3受体介导的基质细胞间相互作用能够促进LUAD微环境重塑和肿瘤侵袭。Notch1受体在LUAD中表现出类似的作用。多项研究^[25,26]^表明，Notch1受体的过度表达与LUAD细胞恶性生物学行为相关。本研究发现，GINS1能够上调Notch1和Notch3受体表达水平促进Notch信号传导，继而磷酸化激活下游PI3K/AKT/mTORC1通路，增强LUAD细胞糖酵解、增殖和转移。并且，本研究通过加入Notch受体激动剂Jagged1多肽和Notch受体抑制剂LY3039478分别进行了回复实验。本研究观察到，在加入Jagged1后，shGINS1-A549组细胞PI3K/AKT/mTORC1通路磷酸化水平升高，糖酵解水平升高，细胞增殖、迁移和侵袭活性均显著提升；而在加入LY3039478后，GINS1-OE-H1299组细胞PI3K/AKT/mTORC1通路磷酸化水平降低，糖酵解水平降低，细胞增殖、迁移和侵袭活性均受到显著抑制。这些结果提示，GINS1在LUAD细胞糖酵解、增殖、迁移和侵袭中表现出的促进作用是由Notch/PI3K/AKT/mTORC1通路介导的。此外，这些结果验证了GINS1与Notch受体之间的直接调控关系，并进一步强调了GINS1-Notch轴与肿瘤细胞糖酵解、增殖、迁移和侵袭之间的联系。

需要指出的是，本研究并未进一步阐明GINS1对Notch受体靶向性调控的具体机制。此外，本研究尚缺乏充足的动物实验进行体内验证。值得强调的是，本研究中体外实验的结论为GINS1与LUAD进展的关系研究提供了一定的参考价值。前文提到，GINS1能够调控DNA复制过程。在未来的研究中，需要重点关注的问题是：GINS1是否直接调控Notch受体编码基因的转录过程?如果是，具体的分子机制如何?这些问题的解决将为GINS1在肿瘤治疗方面的应用提供重要依据。
